# The Amphibians of Mount Oku, Cameroon: an updated species inventory and conservation review

**DOI:** 10.3897/zookeys.643.9422

**Published:** 2017-01-06

**Authors:** Thomas M. Doherty-Bone, Václav Gvoždík

**Affiliations:** 1 Conservation Programmes, Royal Zoological Society of Scotland, Edinburgh Zoo, Edinburgh, United Kingdom; 2 Institute of Vertebrate Biology, Czech Academy of Sciences, Brno, Czech Republic; 3 Department of Zoology, National Museum, Prague, Czech Republic

**Keywords:** Biodiversity, caecilians, Cameroon Volcanic Line, Central Africa, frogs, Lake Oku, montane forests and grasslands

## Abstract

Amphibians are a disproportionately threatened group of vertebrates, the status of which in Sub-Saharan Africa is still uncertain, with heterogeneous fauna punctuated by mountains. Mount Oku, Cameroon is one such mountain, which holds many endemic and restricted-range species. The history of amphibian research on Mt Oku, current knowledge on biogeography and conservation biology is reviewed, including recent findings. This updated inventory adds 25 further species, with 50 species of amphibian so far recorded to the Oku Massif (c. 900 to 3,011 m). This includes 5 endemic to Mt Oku, 7 endemic to the Bamenda Highlands, 18 restricted to the highlands of Cameroon and Nigeria, and 20 with broader ranges across Africa. This includes a new mountain locality for the Critically Endangered *Leptodactylodon axillaris*. Among others, the first record of *Phrynobatrachus schioetzi* and *Ptychadena taenioscelis* from Cameroon are presented. The uncertainty of habitat affinities and elevational ranges are discussed. The proportion of threatened species on Mt Oku is 44.2%, but projected to increase to 47.9% due to new species descriptions and recent dramatic declines. The natural habitats of Mt Oku are irreplaceable refuges for its endemic and restricted-range amphibian populations under severe pressure elsewhere in their range. Threats to this important amphibian fauna are increasing, including agricultural encroachment, expanding aquaculture, livestock grazing, pollution, invasive species, forest loss and degradation. Past, present and desired conservation interventions to address these threats are discussed.

## Introduction

Amphibians are among the most threatened animal group worldwide, with threats ranging from habitat loss, emerging infectious disease, climate change, overexploitation (for food and/or pet trade), invasive alien species and pollution (Stuart et al. 2004; Beebee and Griffiths 2005). Knowledge on the distribution and status of Africa’s amphibians and threats is patchy and generally lags behind the rest of the world (Lawson and Klemens 2001; Schiesari et al. 2007; Gardner et al. 2007; Brito 2008). Cameroon has been comparatively well explored for amphibians in the 20^th^ Century (Amiet 2008). The heterogeneous topography of Cameroon however makes detailing biodiversity distribution difficult.

The very first sampling of amphibians from the Bamenda region was conducted by Lt. Adametz in the early 20^th^ Century (Nieden 1910). However, Mt Oku was apparently not systematically surveyed for amphibians until the 1960s (Amiet 1971). Prior to this study, some amphibians from the general Bamenda Highlands (sometimes referred to as the Bamenda-Banso Highlands) were also sampled by Perret (1966), including the description of *Leptopelis nordequatorialis* Perret, 1966, a species widespread in the highlands of Cameroon and Nigeria. Work was to be followed by Amiet in the 1970s to 1980s, which included the descriptions of many of the restricted range montane species found on Oku. This was followed by the International Council for Bird Preservation (UK) expedition in 1984 (Stuart 1986), which made a preliminary conservation assessment of the herpetofauna of Oku and other mountains in Cameroon (Gartshore 1986). Other herpetologists have subsequently made contributions: an updated inventory was published by Wild (1994); for caecilians (Scolecomorphidae: *Crotaphatrema*) (Nussbaum 1981; Doherty-Bone et al 2011a); Clawed Frogs (Pipidae; *Xenopus*) (Loumont and Kobel 1991; Blackburn et al. 2010a; Evans et al. 2015); Wolterstorff toads (Bufonidae; *Wolterstorffina*) (Perret 1972; Boistel and Amiet 2001); Squeaker Frogs and kin (Arthroleptidae; *Arthroleptis*, *Cardioglossa*) (Blackburn 2008a, b; Blackburn et al. 2010b); Puddle Frogs (Phrynobatrachidae; *Phrynobatrachus*) (Zimkus 2009; Zimkus and Gvoždík 2013).

Here the current knowledge of the amphibians of the Oku Massif is summarised, including results of more recent, mostly unpublished fieldwork by the present authors since 2005. This includes an appraisal of habitats, biogeography, endemic species and their origins. Habitat affinities are reviewed, and the issues affecting their conservation in the near future, including past, present and future interventions to prevent extinctions. Conservation statuses are also proposed for those species newly described. This review and update is expected to act as a primer for more dedicated research and conservation practise on this and other mountains in Cameroon.

## Methods

### Description of study area

**Figure 1.**
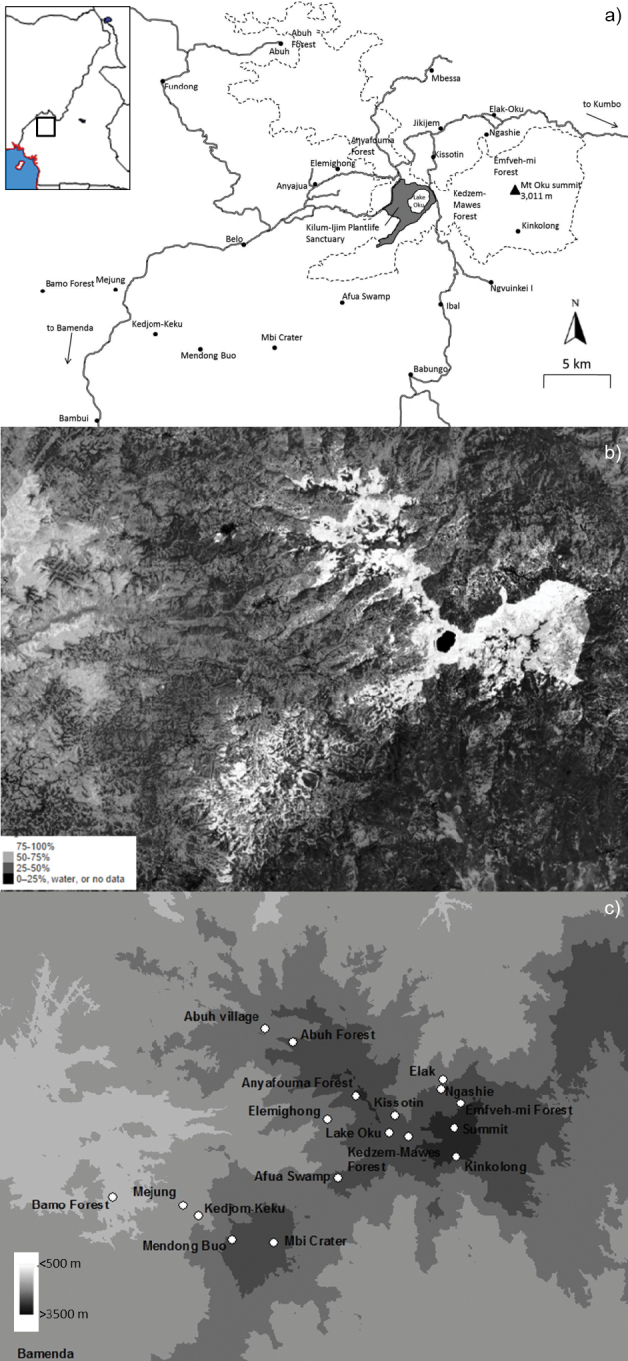
Maps: **a** Main localities on Mt. Oku and surrounding areas (= Oku Massif), including those sampled for amphibians. Dashed line indicates the boundary of the Kilum-Ijim Forest. The grey shaded area shows the Kilum-Ijim Plantlife Sanctuary government protected area. Road (solid lines) bisecting the Kilum-Ijim forest (dashed line) and larger urban centres (Belo, Fundong, Ibal) are also shown **b** forest cover indicated by white shading, from Hansen et al . (2013) (downloaded from https://earthenginepartners.appspot.com/science-2013-global-forest) **c** topographic map showing elevation.

Mount Oku does not form a clear mountain as it occurs on the Bamenda Plateau. Mt Oku is here defined by the boundary of its lower localities within the Oku Massif, such as Big Babanki (1200–1300 m), Bamo Forest west of Big Babanki (900 m), Babungo (1770 m), Ibal (1380 m), Belo (1530 m) and the Mbi Crater (2010 m) (Fig. 1). The term “Mt Oku” used throughout this paper does not exclusively address land controlled by the Oku community, but land also controlled by other communities, such as the Banso, Mbessa, Kedjom-Keku, Fulani and Kom, the latter for example primarily controlling the Ijim Ridge and forest. TMD-B and VG have sampled Elemighong, Anyafouma Forest, Abuh village, Abuh Forest, “Mount Ijim”, Emfveh-mi Forest, Elak-Oku, Kissotin, the Kedzem-Mawes Forest, Afua Swamp, the Mt Oku summit grasslands, Lake Oku and surrounding forest (the Kilum-Ijim Plantlife Sanctuary), Mbi Crater, Bambui, Mendong Buo, Kedjom-Keku village, Mejung village, Bamo Forest (Fig. 1).

Habitats on Mt Oku consist of (as summarised by Forboseh et al. 2003): montane forest; high altitude *Podocarpus* forest; woodland with *Gnidia glauca* and *Maesa lanceolata*; mature bamboo forest; scrubland dominated by *Erica manni*; montane grassland; and subsistence agriculture. Land use in subsistence farms consists of sun crops predominately maize grown with beans, pumpkins and shade crops of coffee, banana, kola nut, avocado, sour plum and other trees. Most farms rear poultry, goats, sheep, pigs and fish (the latter two sometimes combined with pig sheds above fish ponds). Grasslands are often used for grazing of livestock (cattle, sheep, goats).

### Sampling

Amphibian species records from the localities mentioned above were listed from published data and unpublished field studies by the authors. The latter field survey data is presented during the following dates: November to December 2005; June to September 2006; September to December 2008; April to June 2009; November 2009; May 2010; September 2010; May to September 2012; October 2013; December 2014; April 2015. Survey methods during all field seasons typically involved visual and acoustic encounter surveys (searches with surveyor effort recorded) or opportunistic searches (or incidental observations) of habitats during both daylight and night time. Other techniques included pitfall bucket traps (2006 and 2012), dip-netting and aquatic funnel traps in ponds, streams and lakes. Local people also presented animals on an *ad hoc* basis. In addition, TMD-B has recruited and trained local technicians to record observations throughout the year at specific localities (notably Lake Oku) since 2006. In addition to the literature, we re-examined specimens from Mt Oku deposited in the Natural History Museum, London (Collection IDs: *Asytlosternus* – BM1984.356-357; *Phrynobatrachus* – BM1984.445-472; *Xenopus* – BM1984.194-236). Records include tadpoles as well as post-metamorphic individuals.

Threats to the conservation of various species were characterised from IUCN assessments, and from updated observations by the authors, such as incidents of new invasive alien species when encountered or changes in land use. The proportion of threatened species for Mt Oku was calculated following Böhm et al. (2013) by dividing the total species classified as CR, EN, VU by the total number of species already assessed by the IUCN, so excluding DD and Not Evaluated species. For those species that have recently been described, conservation status was proposed based on IUCN criteria (IUCN Standards and Petitions Subcommittee 2016) and the proportion of species threatened on Mt Oku calculated as above.

## Results

**Figure 2.**
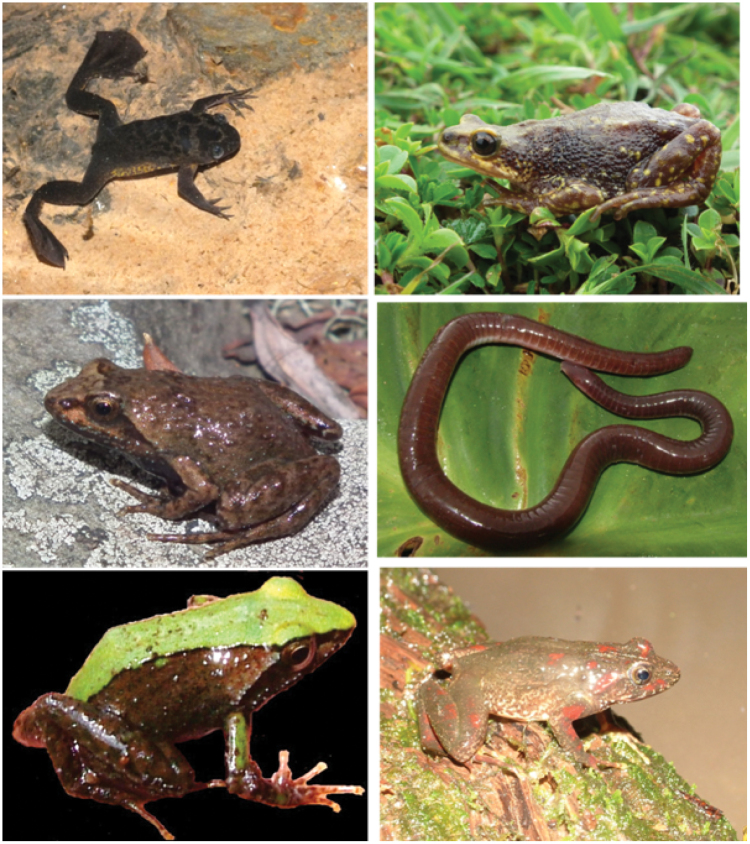
Endemic amphibians of Mt Oku. Clockwise from top-left: Lake Oku Clawed Frog, *Xenopus longipes*; Mount Oku Subalpine Toad, *Wolterstorffina chirioi*; Mount Oku caecilian *Crotaphatrema lamottei*; Lake Oku Puddle Frog, *Phrynobatrachus njiomock* found almost exclusively in forest around Lake Oku; Puddle Frog Phrynobatrachus sp. aff. werneri; Spiny Puddle Frog, *Phrynobatrachus chukuchuku*.

The updated species inventory for Mt Oku found 50 species, of which five are endemic to Mt Oku (pictured in Figure 2), seven endemic to the Bamenda Highlands, 18 restricted to the highlands of Cameroon and Nigeria, 12 with broader ranges across West and Central Africa and eight with Pan-African distributions (Table 1). Contemporary fieldwork added 25 species to the list obtained from published sources, in addition to the proposed new *Phrynobatrachus* discussed below (see Supplemental Table 1 for museum accession numbers). This includes Schiøtz’s Puddle Frog, *Phrynobatrachus schioetzi* Blackburn & Rödel, 2011, representing the first record of this species to Cameroon. This species was described from collections made on the Obudu Plateau, Nigeria from the 1950s and mid 2000s as a possible Nigerian endemic. Cameroonian specimens of this species had been collected on Oku by TMD-B, originally thought to be *Phrynobatrachus werneri* until its description as a valid species in 2011 (Blackburn and Rödel 2011). Further specimens have been observed in Mendong Buo in an open grassland area close to a small forest patch at 2,200 m in 2005, two in Abuh Forest and Elemighong in 2006, two in the Kilum-Ijim Plantlife Sanctuary in 2008, one at the same locality in 2009, two from Afua Swamp in 2009 and one at the Mbi Crater in June 2012. As it has only recently been described, it has yet to be assessed for conservation status by the IUCN. Considering only scarce findings of this species over the years of surveys, but evidently having a wider range as the Obudu and Oku are ca 120 km far from each other, it is likely this species will be listed as Endangered A2(a), B1(b),B2(b): its Estimate of Occurrence (EOO)=1,468 km^2^ and Area of Occupany (AOO)=32km^2^. The identification of *Ptychadena taenioscelis* Laurent, 1954 (specimen accession #: BM2008.476, 16S barcode corresponding to DQ525943, Kenya, Measey et al., 2007) from Afua Swamp is also a new, extreme north-westerly record for this species. Other species new to the published list for Mt Oku include: Arthroleptis cf. perreti Blackburn, Gonwouo, Ernst and Rödel, 2009 (previously referred to as *Arthroleptis variabilis* Matschie, 1893 in Doherty-Bone et al. 2008), *Cardioglossa leucomystax* (Boulenger 1903), *Leptopelis modestus* (Werner, 1898), *Leptopelis notatus* (Peters, 1875), Astylosternus cf. diadematus Werner, 1898, *Astylosternus montanus* Amiet, 1977, *Leptodactylodon axillaris* Amiet, 1971, *Leptodactylodon bicolor* Amiet, 1971, *Trichobatrachus robustus* Boulenger, 1900, *Sclerophrys latifrons* (Boulenger, 1900), *Sclerophrys maculatus* (Hallowell, 1855), *Sclerophrys regularis* (Reuss, 1833), *Sclerophrys superciliaris* (Boulenger, 1888), *Sclerophrys villiersi* (Angel, 1940), *Hyperolius ademetzi* Ahl, 1931, *Hyperolius igbettensis* Schiotz, 1963, *Hyperolius nitidulus* Peters, 1875, Petropedetes sp. aff. parkeri, *Phrynobatrachus calcaratus* (Peters, 1863), *Phrynobatrachus cricogaster* Perret, 1957, *Phrynobatrachus natalensis* (Smith, 1849), Ptychadena cf. mascareniensis “D” (OTU 6; sensu Zimkus et al. 2017) and Ptychadena cf. oxyrhynchus (Smith, 1849).

**Table 1.** A working conservation checklist for the amphibians of Mt. Oku.

Taxon	Species authority	Global IUCN status (those inparentheses are inferred status)	Biogeography	Species Elevational Range (m)		Hypothesised threats	Sources – presence on Mt Oku
				Low	Peak		
Amphibians							
Gymnophiona							
**Scolecomorphidae**							
*Crotaphatrema lamottei*	Nussbaum, 1981	DD	MtO	2175	2398	FL	Nussbaum 1981; Doherty-Bone et al 2011a; Nyingchia and TMD-B pers. ob. 2012, 2013
Anura							
**Arthroleptidae**							
*Arthroleptis adelphus*	Perret, 1966	LC	CWA	900	1788	FL	VG pers. ob. 2005; TMD-B pers. ob. 2006
*Arthroleptis palava*	Blackburn, Gvoždík, Leaché, 2010	LC	CNMts	1400	2200	–	VG pers. ob. 2005, 2009; Blackburn et al. 2010; TMD-B pers. ob. 2006, 2009, 2012
Arthroleptis sp. aff. poecilonotus	–	(LC)	CWA	900	1788	–	Blackburn 2008a; TMD-B pers. ob. 2006, 2012; VG pers. ob. 2005, 2010
Arthroleptis cf. perreti	Blackburn, Gonwouo, Ernst, Rödel, 2009	VU	CNMts	1400	2200	–	VG pers. ob. 2005
*Cardioglossa leucomystax*	(Boulenger, 1903)	LC	CWA	900	1100	FL	VG pers. ob. 2005
*Cardioglossa oreas*	Amiet, 1972	EN	BamH	1900	2650	FL	Gartshore 1986; VG pers. ob. 2005, 2009; Blackburn 2008b; TMD-B pers. ob. 2006, 2010
*Cardioglossa pulchra*	Schiotz, 1963	EN	CNMts	900	1800	–	Gartshore 1986; TMD-B pers. ob. 2006; Blackburn 2008b
*Cardioglossa schioetzi*	Amiet, 1981	EN	CNMts	1640	1800	FL	Blackburn 2006
*Leptopelis modestus*	(Werner, 1898)	LC	CWA	900	2160	FL	TMD-B pers. ob. 2012
*Leptopelis nordequatorialis*	Perret, 1966	LC	CNMts	1000	2000	–	Gartshore 1986; TMD-B pers. ob. 2006, 2009, 2012
*Leptopelis notatus*	(Peters, 1875)	LC	CWA	900	1100	–	VG pers. ob. 2005, 2009
Astylosternus cf. diadematus	Werner, 1898	VU	CNMts	900	1350	–	VG pers. ob. 2005, 2010
*Astylosternus montanus*	Amiet, 1977	NT	CNMts	900	2030	–	TMD-B pers. ob. 2009
*Astylosternus ranoides*	Amiet, 1977	EN	BamH	2200	2600	FL	Amiet 1977; TMD-B pers. ob. 2006, 2008, 2009, 2010, 2012
*Astylosternus rheophilus*	Amiet, 1977	VU	CNMts	1350	2500	FL	Amiet 1977; TMD-B pers. ob. 2006, 2008, 2009, 2010, 2012
*Leptodactylodon axillaris*	Amiet, 1971	CR	BamH	2300	2700	FL	TMD-B pers. ob. 2006
*Leptodactylodon bicolor*	Amiet, 1971	VU	CNMts	950	1788	FL	TMD-B pers. ob. 2006; Ndifon and TMD-B pers. ob. 2015
*Leptodactylodon perreti*	Amiet, 1971	EN	BamH	1500	2650	FL	Amiet 1980; TMD-B pers. ob. 2006, 2009, 2012
*Trichobatrachus robustus*	Boulenger, 1900	LC	CWA	900	1788	OV	TMD-B pers. ob. 2006, 2008; VG, pers. ob. 2010
**Bufonidae**							
*Sclerophrys latifrons*	(Boulenger, 1900)	LC	CWA	900	900	–	VG pers. ob. 2005
*Sclerophrys maculatus*	(Hallowell, 1855)	LC	SSA	900	1788	–	VG pers. ob. 2005; TMD-B pers. ob. 2006, 2008
*Sclerophrys regularis*	(Reuss, 1833)	LC	SSA	900	2500	–	TMD-B pers. ob. 2006, 2008
*Sclerophrys superciliaris*	(Boulenger, 1888)	LC	CWA	900	900	FL	VG pers. ob. 2005
*Sclerophrys villiersi*	(Angel, 1940)	EN	CNMts	1200	2500	FL, SV	VG, pers. ob. 2009
*Werneria bambutensis*	(Amiet, 1972)	EN	BamH	1750	2600	FL, SV	Amiet 1972; VG pers. ob. 2005; TMD-B pers. ob. 2006
*Wolterstorffina chirioi*	Boistel & Amiet, 2001	CR	MtO	3000	3000	CL, SV	Boistel and Amiet 2001; Nyingchia and TMD-B pers. ob. 2012
*Wolterstorffina mirei*	(Perret, 1971)	EN	BamH	1800	2800	FL, SV	Boistel and Amiet 2001; Nyingchia and TMD-B pers. ob. 2008, 2009, 2012
**Hyperoliidae**							
Afrixalus “quadrivittatus”﻿	Pickersgill, 2007	LC	SSA	900	1788	–	TMD-B pers. ob. 2006, 2008, 2012; Amiet 2009
*Hyperolius ademetzi*	Ahl, 1931	NT	CNMts	900	2220	–	VG pers. ob. 2005
*Hyperolius igbettensis*	Schiotz, 1963	LC	CWA	900	2010	–	TMD-B pers. ob. 2012
*Hyperolius nitidulus*	Peters, 1875	LC	CWA	900	2010	–	TMD-B pers. ob. 2012
*Hyperolius riggenbachi*	(Nieden, 1910)	VU	CNMts	1100	2010	–	Gartshore 1986; VG pers. ob. 2005, 2009, 2010; Khimal and TMD-B pers. ob. 2006, 2008, 2009, 2012
*Kassina maculosa*	(Sternfeld, 1917)	LC	CWA	900	2600	–	Amiet 2007; VG pers. ob. 2005; TMD-B pers. ob. 2009
**Petropedetidae**							
Petropedetes sp. aff. parkeri		(DD)	CNMts	1090	1300	–-	VG pers. ob. 2005
**Phrynobatrachidae**							
*Phrynobatrachus calcaratus*	(Peters, 1863)	LC	CWA	900	1200	FL	VG pers. ob. 2005
*Phrynobatrachus chukuchuku*	Zimkus, 2009	CR	MtO	2230	2800	SV, CL	Zimkus 2009; TMD-B pers. ob. 2012
*Phrynobatrachus cricogaster*	Perret, 1957	VU	CNMts	900	1850	FL	VG pers. ob. 2005
*Phrynobatrachus jimzimkusi*	Zimkus, Gvoždík, Gonwouo, 2013	(CR)	CNMts	*c.*1300	*c.*2800	FL	Gartshore 1986; Zimkus and Gvoždík 2013; VG pers. ob. 2005, 2009; TMD-B pers. ob. 2006, 2008, 2009, 2010
*Phrynobatrachus natalensis*	(Smith, 1849)	LC	SSA	900	2200	–	TMD-B pers. ob. 2006
*Phrynobatrachus njiomock*	Zimkus & Gvoždík, 2013	(CR)	MtO	2200	2400	FL, IN	Gartshore 1986; Zimkus and Gvoždík 2013; Amiet 1978; VG pers. ob. 2005; Zimkus 2009; TMD-B pers. ob. 2006–2010
*Phrynobatrachus schioetzi*	Blackburn & Rödel, 2011	(VU)	CNMts	1500	2200	FL	Hořák and VG pers. ob. 2005; TMD-B pers. ob. 2006, 2008, 2009, 2012
*Phrynobatrachus steindachneri*	Nieden, 1910	VU (CR)	CNMts	1300	2460	FL	Amiet 1971; Gartshore 1986; Zimkus 2009; Zimkus and Gvoždík 2013; TMD-B pers. ob. 2006, 2009
*Phrynobatrachus werneri*	(Nieden, 1910)	LC (VU)	CNMts	1200	2200	–	Gartshore 1984 (from BMNH collection, not reported in 1986 paper); TMD-B pers. ob. 2006, 2009, 2012
Phrynobatrachus sp. aff. werneri	–	(CR)	MtO	*c.*2000	*c.*2800	FL	TMD-B pers. ob. 2006, 2008, 2009
**Pipidae**							
*Xenopus eysoole* (previously referred to as *Xenopus amieti*)	Evans, Carter, Greenbaum, Gvoždík, Kelley, McLaughlin, Pauwels, Portik, Stanley, Tinsley, Tobias & Blackburn, 2015	(NT)	BamH	1100	2175	–	Loumount and Kobel 1991; Gartshore 1986; Evans et al. 2015; Tropek and VG pers. ob. 2009; TMD-B pers. ob. 2006, 2008, 2009, 2012, 2015
*Xenopus poweri* (previously referred to as *Xenopus laevis*)	Hewitt, 1927	LC	SSA	900	1788	–	Loumount and Kobel 1991; TMD-B pers. ob. 2006
*Xenopus longipes*	Loumount & Kobel, 1991	CR	MtO	2220	2220	IN, FL	Gartshore 1986; Loumount and Kobel 1991; Tinsely and Measey 2004; VG pers. ob. 2005; Blackburn et al. 2010; Doherty-Bone et al. 2013b; TMD-B, pers. ob. 2006–2015
**Ptychadenidae**							
Ptychadena cf. mascareniensis “D” (OTU 6; sensu Zimkus et al. 2017)	–	LC	SSA	900	*c.*2000	–	VG pers. ob. 2009; TMD-B 2006, 2009
Ptychadena cf. oxyrhynchus	(Smith, 1849)	LC	SSA	900	*c.*2000	–	VG pers. ob. 2010
*Ptychadena taenioscelis*	Laurent, 1954	LC	SSA	900	2029	–	TMD-B pers. ob. 2009

Abbreviations for IUCN Conservation Status: CR – Critically Endangered ; EN – Endangered ; VU – Vulnerable ; NT – Near Threatened ; LC – Least Concern ; DD – Data Deficient . Elevation range is provided for the species population living on Mt Oku. Abbreviations for biogeography: SSA – widespread throughout sub-Saharan Africa ; CWA – restricted to Central and West Africa countries, but still widespread ; CNMts – restricted to the highlands of Cameroon and Nigeria ; BamH restricted to the Bamenda Highlands, including Mt. Mbam, Mt. Lefo and Bamboutos Mts.; MtO restricted to Mt. Oku Abbreviations for hypothesised threats: FL – forest loss and degradation ; SV – threats to savanna species, including overgrazing and fire ; CL – Climate Change ; OV – Overexploitation ; IN – Introduced Species , all inferred based on IUCN assessments and field observations of the authors. Threats from pollution and disease are unknown.

Should one *Phrynobatrachus* species be confirmed to be new to science, this would make this 51 species to the mountain, of which 6 will be endemic. This potentially new, undescribed, endemic puddle frog, Phrynobatrachus sp. aff. werneri is here presented – this morpho-species is similar to *Phrynobatrachus werneri* (Nieden, 1910) and has not been observed on other mountains or in other collections. It is similar to *Phrynobatrachus werneri*, but is more gracile like *Phrynobatrachus chukuchuku* Zimkus, 2009 and has a more pointed snout (Fig. 2). Phrynobatrachus sp. aff. werneri varies in colouration, with green or yellow bands or a narrow-yellow stripe down the centre of the back. As it has only been observed exclusively in forest with larger trees in Anyafouma Forest and Abuh Forest, it is proposed that it could be threatened by forest loss. It has also never been recorded >500m from the forest edge. It is represented by only one voucher specimen, but has not been observed on Mount Oku since 2009 for further collections to be made to create an appropriate type series to describe it. Following the assessment criteria of the IUCN, the extremely limited range (EOO is negligible, AOO=8 km^2^) and sustained decline of this species would qualify it as Critically Endangered A2(a,b) B2(b) (IUCN Standards and Petitions Subcommittee 2016).

The calculated proportion of threatened species (assuming data deficient and not evaluated species will have an equivalent distribution of conservation status to threatened species, Böhm et al. 2013) based on the recorded species (excluding Phrynobatrachus sp. aff. werneri) was 44.2%. Of the 50 species, 19 amphibian species have already been assessed to be threatened with extinction at different intensities: seven Vulnerable; eight Endangered; four Critically Endangered (Table 1). Hypothesised threat assessments to all species predominately include: forest loss (23 out of 50 species); burning and overgrazing of grassland (four species); one case of overexploitation (Hairy Frogs, *Trichobatrachus robustus*); two cases of invasive species (*Xenopus longipes* Loumont & Kobel, 1991 and possibly *Phrynobatrachus njiomock* Zimkus & Gvoždík, 2013 threatened by exotic fish); and two possible cases of climate change being a threat (for the Mt Oku Subalpine Toad, *Wolterstorffina chirioi* Boistel & Amiet, 2001 and Spiny Puddle Frog, *Phrynobatrachus chukuchuku*). Threats that have not been appraised for these species include pollution and susceptibility to disease.

Of the newly described species so far un-assessed by the IUCN, it is proposed *Phrynobatrachus njiomock* be given a classification of Critically Endangered (IUCN criteria: A2(a),B2(b)) due to its extremely limited range size (EOO=12 km^2^, AOO=20 km^2^) within and around Lake Oku. It has also not been observed since 2010 despite monthly monitoring at Lake Oku and the Oku summit. It is also proposed that both *Phrynobatrachus jimzimkusi* Zimkus, Gvoždík & Gonwouo, 2013 and *Phrynobatrachus steindachneri* Nieden, 1910 be given a classification of Critically Endangered A2(a), A4(a), B1(b), B2(b) due to their limited range sizes and recent, substantial declines (Hirschfeld et al. 2016). *Phrynobatrachus jimzimkusi* has been unobserved on Mt Oku since 2010, *Phrynobatrachus steindachneri* since 2009. *Phrynobatrachus werneri*, while observed in agricultural areas, has also become rare, from high sample success rate of several individuals in 2006 and 2009 to no individuals or rare occurrences of singletons at those same localities from 2010 onward (Hirschfeld et al. 2016, TMD-B pers. obs.). It may thus be inferred that this species would be downgraded from Least Concern to Vulnerable A2(b),B1(b),B2(b) due to its decline at least on Oku and restricted range to the Highlands of Cameroon and Nigeria. If it has declined elsewhere across its range as on Oku and Manengouba, it might be downgraded further to Endangered. The newly described *Xenopus eysoole* Evans, Carter, Greenbaum, Gvoždík, Kelley, McLaughlin, Pauwels, Portik, Stanley, Tinsley, Tobias, Blackburn, 2015) show similar habitat affinities to its original synonym *Xenopus amieti* Kobel, du Pasquier Fischberg & Gloor, 1980 in that it occurs almost exclusively in open areas, including in agriculture but restricted to elevations above 1100 m a.s.l. and thus vulnerable to climate change and possibly increases in use of agrochemicals (see below). It may thus be assessed to be Near Threatened by the IUCN criteria. These proposed changes to IUCN status discussed above would increase the proportion of threatened amphibian species on Mt Oku to 47.9%. If Phrynobatrachus sp. aff. werneri is indeed a valid species, this would make the proportion of threatened species 48.9%.

## Discussion

Intensive field work over the past 10 years was combined with published records on the amphibian species present on Mt Oku. One hundred years of surveys have yielded 50 species, of which nearly half have been recorded in the past 10 years. This has brought together knowledge to make improved research, monitoring and conservation planning possible. A significant proportion of these frogs were found to be threatened, and the intrinsic and extrinsic causes of this are discussed below.

### Biogeography and Origin of Mount Oku’s Amphibians

The amphibian fauna of Oku appear to share an affinity with nearby localities such as Mt Mbam, Mt Lefo and Bamboutos Mts, and sharing same restricted range and Bamenda Highland endemic species on Mt Tchabal Mbabo (Cameroon), Gotel Mountains (Cameroon – Nigeria) and Mambilla Plateau (Nigeria). For example, all firstly listed three peaks share species such as *Astylosternus ranoides* Amiet, 1977, *Cardioglossa oreas* Amiet, 1972 and the restricted range *Astylosternus rheophilus* Amiet, 1977. Oku and Bamboutos share *Werneria bambutensis* Amiet, 1972 and *Wolterstorffina mirei* Perret, 1971 (Perret 1972), while Oku and Tchabal Mbabo share very similar *Crotaphatrema* caecilian species, *Crotaphatrema lamottei* and *Crotaphatrema tchabalmbamboensis* Lawson, 2000 (Doherty-Bone et al. 2011a), and two lineages of the *Phrynobatrachus steindachneri* species complex (Zimkus and Gvoždík 2013). An adequate, robust appraisal (such as an analysis of similarity indices across the range) of the biogeography of Oku’s amphibians is difficult due to: a) the absence of complete inventories at other localities around Cameroon and b) the turbulent taxonomy of Cameroon’s amphibians over the last decades.

Understanding uniqueness and therefore potential recovery options (such as translocation) for Mt Oku’s threatened amphibians requires an adequate appraisal of their evolutionary history. This is however not robustly possible at present due to: a) incomplete distribution and availability of genetic data; and b) an apparently low research effort on this topic. Evolutionary questions to address origins of some amphibian genera within the African continent have been investigated but few have addressed Mt Oku specifically. Exceptions include the discussion of speciation in *Cardioglossa* (Blackburn 2008) and hypothesis on the polyploid speciation and origin of *Xenopus* (Evans et al. 2015). The speciation of Oku’s amphibian species is currently explained by the isolation of Oku from other mountains during Pleistocene climatic changes when habitats became fragmented (Amiet 2008). However, the relatively few Mt Oku endemic and numerous Bamenda Highland endemic species in relation to other northern mountains is consistent with phylogenetic evidence from certain avian species (Smith et al. 2000), suggesting the higher plateau of the “northern mountains” (Bamboutos, Oku, Mbam, Mambilla Plateau, Gotel Mts, Tchabal-Mbabo) had greater connectivity during cold and dry climatic phases. The phylogeny of the Cameroonian *Phrynobatrachus* and phylogeography of the *Phrynobatrachus steindachneri* complex support hypotheses about pre-Pleistocene radiation and speciation events probably in connection to the orogeny and rich volcanic activities during the Late Tertiary (Zimkus and Gvoždík 2013).

### Mount Oku Endemics

Endemic species of Mount Oku include the Lake Oku Clawed Frog, *Xenopus longipes* restricted to Lake Oku, the Mount Oku Subalpine Toad, *Wolterstorffina chirioi* restricted to the subalpine grasslands on the Mount Oku summit. The Spiny Puddle Frog, *Phrynobatrachus chukuchuku* also found only in the high elevation grasslands of the Oku summit. It is more abundant than *Wolterstorffina chirioi*, and has been found as far from the summit as the wetland at Kinkolong (2710 m a.s.l.). A tadpole barcoded with a matching haplotype has however been recorded at Abuh at a stream at the forest-grassland boundary (2,160 m a.s.l.) (Pfalzgraff et al. 2015), suggesting it may have a broader distribution but still restricted to montane grassland. It has recently been assessed to be Critically Endangered by the IUCN due to its restricted range and grazing pressure of its limited habitat (IUCN SSC Amphibian Specialist Group 2011). Here it is hypothesised that climate change may also threaten this species, as similarly hypothesised to threaten the sympatric *Wolterstorffina chirioi* (IUCN SSC Amphibian Specialist Group 2015a). The Puddle Frog, *Phrynobatrachus njiomock* was recently formally described (Zimkus and Gvoždík 2013) but was previously known as *Phrynobatrachus* sp. 11 (sensu Amiet 1978) – this frog has also been referred to as Phrynobatrachus cf. steindachneri (Doherty-Bone et al. 2008, 2013b) and resembles *Phrynobatrachus steindachneri* and *Phrynobatrachus jimzimkusi* which also occur on Mt Oku. This species is mostly restricted to the forest adjacent to Lake Oku, with some individuals found towards the summit.

The Mount Oku Caecilian, *Crotaphatrema lamottei* Nussbaum, 1981 – a very rare, limbless burrowing amphibian has only been recorded to Mt Oku. A similar species, *Crotaphatrema tchabalmbaboensis* occurs on Mt Tchabal Mbabo, Adamawa Region, ca. 230 km north-east. A recent study found these two species to have a very low genetic difference, suggesting they could be conspecific, possibly treated as different subspecies, but requires further examination of more specimens and molecular markers (Doherty-Bone et al. 2011a). It is possible that this caecilian is more widespread over the mountains (probably including the Nigerian Mambilla Plateau and Gotel Mts) but overlooked. The Oku community consider contact with this worm-like amphibian to be bad-juju (bringer of bad luck) and require a traditional medicine man to provide a cleansing potion on the event of digging up, touching or accidentally killing one (Doherty-Bone et al. 2011b). As it is still inconclusive whether or not this species is dependent on forest or if it is threatened by agriculture, it remains listed as Data Deficient by the IUCN.

The potential undescribed endemic puddle frog, Phrynobatrachus sp. aff. werneri has not been observed since 2009 in Anyafouma Forest, despite searches in 2012 to present. This makes taxonomic appraisals difficult as it is represented by only a single voucher specimen. So far it has not been observed on other mountains, including in the Bamenda Highlands. Careful appraisal of the taxonomy of this species is now needed in the absence of additional specimens, should no further individuals be observed. If individuals are observed, careful consideration should be put into collection of voucher specimens from what could be a recovering population of a Critically Endangered species (Minteer et al. 2014).

### Bamenda Highlands Endemics

There are seven species that occur on Mt Oku which also occur on nearby mountains in the Bamenda Highlands, such as Mts Bamboutous, Lefo, Santa, and Mbam. These include Perret’s Egg Frog, *Leptodactylodon perreti* Amiet, 1971 – The type specimen for this species was collected from Mt Mbam, with other populations found on Mt Oku. It can be found in most forests on Mt Oku, though it is difficult to find individuals, even when among a chorus. Their choruses have been heard from near the farm-forest boundary by Elak-Oku, to the woodland just below the Oku summit grassland. It has apparently never been observed outside of forest, and is probably a forest-dependent species. It has been listed as Endangered by the IUCN due to its restricted range and predicted vulnerability to forest loss. Another Egg Frog corresponding to *Leptodactylodon axillaris* has recently been found to occur on Mt Oku. This species was previously only recorded to Mt Bamboutos, and has thus been assessed to be Critically Endangered (IUCN SSC Amphibian Specialist Group 2013). The population corresponding to this species should be verified as to whether it is con-specific to those on Mt Bamboutos, given the potential for high elevation frogs to have cryptic species. Should con-specific status between the two mountains be confirmed, extinction of this species would be less imminent given the severe threats on Bamboutos.

The Bamboutos Small-tongue Toad, *Werneria bambutensis* type locality is Mt Bamboutos. There have been no individuals observed on Oku since 2006 (by TMD-B) where it was rare: two were presented by local women in Elemighong who were out farming (but distance to forest was not clear), another captured in grassland near Anyafouma Forest, suggesting it is not entirely dependent on forest. The requirements of its full life-cycle are not clear however. Tadpoles were found by VG in a stream in a small forest patch at 2,100 m a.s.l. in the beginning of dry season (late November 2005). It is listed as Endangered by the IUCN because of its restricted range and threat from forest loss (Amiet 2004a).

The Mount Oku Long-fingered Frog, *Cardioglossa oreas* also occurs on Mt Bamboutos and Mt Lefo. Mt Manengouba has been cited as a locality for this species, but these reports have been attributed to its sister species *Cardioglossa manengouba* Blackburn 2008 prior to the latter’s description (Blackburn 2008b). It has been collected in a forest patch above Big Babanki (VG), high elevation grassland ~20 m from Anyafouma Forest boundary and at Lake Oku (TMD-B, VG) and other reports of an affinity with bamboo forest and streams (Gartshore 1986). It has been listed by the IUCN as Endangered, as it is thought to be dependent on forest (IUCN SSC Amphibian Specialist Group 2015b). Indeed *Cardioglossa oreas* has never been found far (< 500 m) from the boundary of primary forest blocks. However, it seems that this species may survive even in small forest patches, which might serve for its dispersal across a fragmented landscape (VG, personal observation), and should be assessed further.

Other Bamenda Highland endemic species include the Mount Oku Wolterstorff Toad, *Wolterstorffina mirei* – Mt Oku is the type locality for this species, and is found also on Bamboutos (Perret 1972). These toads are quite rare on the mountain, found in both forest and higher savanna. The Central Night Frog, *Astylosternus ranoides* type locality is Mt Bamboutos. On Oku, it can consistently be observed around most streams in or near forest, particularly around Lake Oku. It is rarely found outside the forest and is likely a forest dependent species. It is one of the few montane endemic frog species to have not experienced a population crash as observed for other sympatric species on Mt Oku (Hirschfeld et al 2016).

### Restricted-range mountain specialists

Restricted-range mountain specialists include Steindachner’s Puddle Frog, *Phrynobatrachus steindachneri*, previously found in forests and forest openings throughout the Kilum-Ijim Forest, where it appeared to be the dominant leaf litter anuran together with similar, closely related *Phrynobatrachus jimzimkusi* (Zimkus and Gvoždík 2013). *Phrynobatrachus steindachneri* is also recorded on mountains in western Adamawa Region, Cameroon, and eastern Nigeria, while *Phrynobatrachus jimzimkusi* is distributed from Mt Oku southward to Mt Manengouba and westward to the Obudu Plateau in Nigeria (Zimkus and Gvoždík 2013). *Phrynobatrachus jimzimkusi* has been found in more deforested areas, such as in home-gardens in Elak-Oku, so could be more tolerant to forest loss. Both species have been previously lumped under the name *Phrynobatrachus steindachneri*, which is currently listed as Vulnerable by the IUCN due to the threat from forest loss and degradation. As both species have sustained long term (since 2010) declines on Mt Oku (Hirschfeld et al. 2016; TMD-B pers. ob.), it is proposed they are more threatened than previously thought, and may require more severe conservation classification (see above).

Werner’s Puddle Frog, *Phrynobatrachus werneri* was common in most habitats on Oku, including both primary montane forest and agricultural areas. It is restricted to the highlands of Cameroon and Nigeria, and is assessed to be Least Concern by the IUCN. As with other *Phrynobatrachus* in the highlands of Cameroon, it was once abundant (especiallly in Elemighong) but has undergone a dramatic and not easily explained decline in abundance (Hirschfeld et al. 2016), though a single specimen was found in Afua Swamp and another at the Mbi Crater in 2012 by TMD-B.

Other Cameroon highland species with broader ranges include the Cameroon Range Night Frog, *Astylosternus rheophilus* – found particularly in agricultural areas, but also in forest. This species is endemic to Cameroon, though may occur in Nigeria. It is found also on Mt Bamboutos, Mt Lefo, and on Tchabal Mbabo from where a separate subspecies was described (*Astylosternus rheophilus tchabalensis* Amiet, 1977). It has been listed as Vulnerable by the IUCN as it could be threatened by habitat degradation, though it has a larger range than other similar *Astylosternus* species (Amiet 2004b).

Riggenbach’s/Hieroglyphic Reed Frog, *Hyperolius riggenbachi* (Nieden, 1910)– This species has been observed as high as Elak-Oku (1960 m a.s.l.), and also collected at the Mbi Crater and Elemighong. It appears to thrive in agricultural settings and can be one of few species observed in most degraded areas. It has been listed as Vulnerable by the IUCN due to its restricted range, though it does not seem to be threatened by forest loss. Its resilience to agrochemicals and intensified land use is not known.

The Bamenda Reed Frog, *Hyperolius ademetzi* is endemic to the highlands of Cameroon and is found as far south as Mt Manengouba. This is the first report of this species occurring on Mt Oku, where it was observed by VG in the Kilum-Ijim Plantlife Sanctuary by Lake Oku. As it occurs mostly in grasslands, the IUCN has assessed it to be Near Threatened. Similarly, the Tree Frog, *Leptopelis nordequatorialis* is also found in open areas, including cultivated areas and swamps. It has been collected in Elemighong and Afua Swamp. It is restricted to the highlands of Cameroon and Nigeria, and has been assessed to be Least Concern by the IUCN. The extent of the resilience of these species to grazing, grassland burning, agrochemicals and intensification of land use is not understood.

A common pipid frog has been observed in the Oku Massif and had been previously referred to as *Xenopus amieti* (Loumont and Kobel 1991). However, frogs assigned to this species on Mt Oku have recently been assigned to a separate species, *Xenopus eysoole* (Evans et al. 2015). It is common throughout Oku and Bamenda Highlands. It is found in most disturbed habitats, strangely never in forest, but this could be explained by a preference for still waters and open areas, as opposed to the shaded, lotic habitats that dominate forests on Oku. The current range of *Xenopus amieti* is now uncertain with regard to whether it is sympatric or allopatric to *Xenopus eysoole*, with updated surveys needed to enable more accurate assessment of their conservation status.

### Habitat and elevation affinities of Mount Oku’s amphibians

Mt Oku’s landscape has historically been altered by the demand for fuel wood, grazing of livestock, encroaching agriculture, extirpation of many larger animals, fire and possibly even climate change (Cheek et al. 2000). Understanding the habitat requirements of Oku’s amphibians is therefore challenging due to “ghost of land use past” (*sensu* Harding et al. 1998), but critical for predicting their future population trends and planning appropriate action such as habitat protection. Habitat affinities are discussed rather than actual habitat requirements of Mt Oku’s amphibians, as a consistent study on habitat requirements has yet to take place. Instead, data on which habitat each species has been observed is available (Gartshore 1986 and listed on the IUCN Red List: www.iucnredlist.org and references therein), but does not robustly predict consequences of habitat change or take elevation distribution into account. These consist mostly of anecdotal, expert opinions and/or surveys that suffer from design issues, such as pseudoreplication, confounding edge effects and the lack of adequate, clearly defined control sites (Gardner et al. 2007). Addressing these issues is challenging due to the physical terrain and size of the study area. Identifying a pristine control site that represents a large block of undisturbed forest is also a challenge as most of the Kilum-Ijim Forest has been degraded, including the Plantlife Sanctuary. The latter is perhaps the most appropriate candidate for a control site, along with the sacred forest by Elak-Oku, which local elders claim is among the least disturbed in the Kilum (Oku) forest.

Emerging evidence suggests that most of the endemic and Bamenda Highland endemic species rely on forest on Mt Oku (see above and Table 1) and two endemic species occur exclusively in high elevation grasslands, though whether they are dependent on grassland or the climatic envelope montane grassland occurs at is not known. All these habitats are threatened by livestock grazing, which appears to be increasing in forests, with high densities on the grasslands. Some endemic species have very specific habitat affinities, such as *Phrynobatrachus njiomock* being predominantly recorded in the forest around Lake Oku, and the Lake Oku Clawed Frog (*Xenopus longipes*) being restricted to Lake Oku. Differentiating affinities for habitat from affinities for particular elevations is challenging for those species that dwell at high elevations above 2,000 m a.s.l.. Forest-dependent species that are adapted to mid-elevations will be difficult to identify as most forest has been lost at mid-elevations, and any such species would be either locally extinct, occur at low abundance or occur in fringe habitat that obscures its complete elevational range. Resolving these issues will require consistent sampling on nearby mountains with varying deforestation histories at set elevations (i.e. 1,000 m, 1,500 m, 2,000 m, etc.), at least for those species shared by other mountains.

### Conservation of Mount Oku’s Amphibians

As with the rest of the world and Cameroon (i.e. Beebee and Griffiths 2005; Amiet 2008), issues affecting amphibians are effectively the same affecting the wider environment on Mt Oku. The loss and degradation of forests, chemical pollution, invasive species (including pathogens) and climate change all threaten the unique biodiversity of Oku as much as it threatens its amphibians. Predicting the specific threats is however difficult due to the paucity of robust ecological data. Amphibians require freshwater breeding habitats, as well as terrestrial habitats to forage. These also require physical conditions to allow eco-physiological persistence, such as moisture (when on land), low salinity and conditions to avoid predation (freedom from fish or cover from birds) (as reviewed in Smith and Sutherland 2014). It is therefore possible to pre-emptively hypothesise threats in absence of robust data that could be obtained at a later date. Certain amphibian populations have however received consistent, specific survey attention, such as the Lake Oku Clawed Frog (*Xenopus longipes*), leaf-litter forest anurans, farmland species and more recently anurans of the high grasslands of the Oku summit. Below we discuss other threats to amphibians on Mt Oku, notably disease, climate change, pollution and invasive species.

### Forest degradation and loss

Mt Oku has been inhabited by people for hundreds of years and it has been estimated that widespread deforestation occurred through clearance for agriculture during the rise of the centralised agrarian societies that are now the fondoms (= kingdoms) (Cheek et al. 2000). During the colonial era, the remaining forest on the top of Mt Oku was protected as a forest reserve, but following independence the new government forestry department allowed the forest to decline. Despite renewed protection measures for the Kilum-Ijim forests (see below), the forest is likely to continue to degrade due to edge-effects, extirpation of seed-dispersing birds and mammals (e.g. Maisels et al. 2001), incursions by livestock, likely climate change, chronic over-use by the local community and infrastructure developments.

Forest management is by local community forest management institutes (FMIs), with the exception of the government controlled (Ministry of Forestry and Wildlife) Kilum-Ijim Plantlife Sanctuary which includes Lake Oku. Forests are accessed extensively by the communities for placing bee hives, collection of medicinal plants, hunting and passage to other villages. Firewood is frequently collected from the forest: the FMI’s have declared that only wood from dead trees be collected from the forest, but in recent years live trees cut down have been increasingly observed, as has the killing of trees through debarking (Stewart 2009; TMD-B, pers. ob.).

More recently, a section of forest was cut down in 2012 to make way for an ambiguous government funded tourism development near (though not immediately adjacent to) Lake Oku. This has apparently set a precedent for more forest clearance for development in 2015 with the Cameroon Baptist Convention clearing another patch of forest adjacent to the aforementioned site, to create a new church and a series of dormitories (TMD-B, pers. ob.). Should they become successful in attracting customers (no plans exist for low impact ecotourism), this is likely to result in further demand for fuel wood and general disturbance of the forest. Roads passing through the forests have recently been improved and are likely to increase access to the forest to extract resources such as bushmeat and fuelwood.

### Livestock grazing

Livestock are found to regularly make incursions into the forest (pers. ob.). Livestock grazing has historically been on the increase, especially in the Summit grasslands (Maisels et al. 2000; Doherty-Bone 2015a) but also on most high elevation grasslands, such as by Anyafouma and Abuh. These often co-occur with incursions into the forests. All these practises are novel to these ecosystems, though the extent to which grazing by locally extinct larger mammals is replaced by domestic livestock is unknown. Livestock overgrazing represents a threat to amphibians through altering habitat by removal of vegetation, compacting soils, causing erosion into aquatic habitats, and restricting recruitment of native trees. This is in combination with burning by pastoralists in an attempt to provide better quality browse for animals, which would directly kill amphibians and alter their habitats.

### Exploitation

Amphibians in Cameroon are in some areas commonly harvested for human consumption (Gonwouo and Rödel 2008). In the highland areas at Babanki, larger species of frog such as Hairy Frogs (*Trichobatrachus robustus*) and tadpoles of *Astylosternus* and *Trichobatrachus* are often consumed (VG pers. ob.). Collection of amphibians for the commercial pet trade apparently also occurs from Cameroon (based on animals presented for sale in Western countries claimed to originate from Cameroon, Herrel and van der Meijden 2014). However, the authors have not observed animals being collected for trade, or heard about brokers organizing local people to collect animals on Mt Oku so far, unlike elsewhere. There have been isolated incidents when Western animal dealers have contacted Cameroonian field assistants about acquiring species such as *Xenopus longipes*. It was not clear how these dealers obtained the contact details of these field assistants, but these appeared to be prospective inquiries rather than trade *per se*. This is still activity that professional herpetologists should be aware of when training local guides and sharing their contact details.

### Chemical pollution

The role of chemical pollution is an old topic in amphibian conservation biology (e.g. Rouse et al. 1999), but Africa has received an especially low research effort (Schiesari et al. 2007). Industry and use of agrochemicals is growing in Cameroon. Mt. Oku is still isolated from the effects of factories and is generally not subjected to industrial agriculture, with the exception of a tea estate adjacent to the Mbi Crater at Ndawara. However, local farmers are increasingly using agrochemicals for their crops, such as glyphosate herbicides that are on sale in many shops in villages. Other sources of pollution on Mt Oku include an increasing incidence of plastic refuse, electrical waste (especially batteries) and the growing number of vehicles in the area. Garbage is often found around the one section of lake that is visited by tourists and locals alike. Three roads bisect the Kilum-Ijim forest (Figure 1) and traffic has been noticed to increase in recent years following improvements to these roads (TMD-B. pers. obs.). Other forms of pollution include elevated UV-radiation from ozone depletion, which impacts certain species of northern hemisphere amphibians in the laboratory (Blaunstein et al. 1994), but unknown for African amphibians, particularly those that occur at high elevations, such as on Oku. Responses of Mt Oku’s amphibians to these multiple and growing sources of chemical pollution are unknown and warrant further investigation, which could include surveys on farms using particular chemicals, and toxicity experiments on target species to field-relevant agrochemical mixtures.

### Climate change

Tropical mountains are predicted to undergo dramatic ecological changes as a result of global climate change, including changes to temperature and precipitation (Beniston et al. 1996). In Cameroon, temperatures have been increasing since at least 1960, with changes to rainfall predicted to increase incidents of drought (Molua 2006). This has the potential to impact the amphibian assemblage of Mt Oku. Firstly, there may be a shift in distribution based on elevation as temperatures change, with endemic species at higher elevations moving even higher, reducing their range size (as observed in Madagascar, Raxworthy et al. 2008). Another effect will be changes to hydrology associated with changes in precipitation. This could lead to potentially reduced availability of streams and ponds for breeding, affecting both breeding phenology and density of amphibians across habitats. Further consequences of this could include increased competition between species and increased transmission of disease. Other consequences of increased drought will be a decline in invertebrates, reducing the prey-base for amphibians (Donnelly and Crump 1998). High elevation endemic species hypothesised to be most threatened by climate change on Mt Oku are the Subalpine Toad, *Wolterstorffina chirioi* (Boistel and Amiet 2001), with the sympatric Spiny Puddle Frog, *Phrynobatrachus chukuchuku* possibly also threatened due to its restricted climatic-range. It is however likely there are many more amphibians on Mt Oku threatened by climate change.

### Disease

Disease is a major issue in the conservation of amphibians worldwide (Dazak et al. 1999; Skerratt et al. 2007; Duffus 2009). The amphibian-killing chytrid fungus (*Batrachochytrium dendrobatidis - Bd*) for example has caused declines of many amphibian species. Cameroon has one of the oldest records for this pathogen, recorded in a frog collected from eastern lowland Cameroon in 1933 (Soto-Azat et al. 2010). A 2006 survey of amphibians on Mt Oku found no evidence for the presence of this pathogen (Doherty-Bone et al. 2008) but subsequent sampling has found it to be present at a prevalence comparable to other regions of Africa (Doherty-Bone et al. 2013a; Hirschfeld et al. 2016). However, the role of *Bd* in amphibian population viability in Cameroon is still cryptic due to new findings that multiple strains of varying virulence occur within Africa (Farrer et al. 2011). While *Bd* is hypothesised to originate from Africa (Weldon et al. 2004), it has more recently been associated with declines of amphibians in the Highlands of Cameroon, including Mt Oku where it had not been detected prior to declines (Hirschfeld et al. 2016). Sequencing of strains of this fungus from localities in Cameroon is now a priority to understand if strains are from hyper-virulent or benign variety (one sample was said to be from the pan-global hypervirulent strain, but was not accompanied by peer-reviewed data in Hirschfeld et al. 2016). Using archived museum specimens to determine the long-term history of this pathogen on Mt Oku will test the endemic versus novel pathogen hypothesis in Cameroon (Soto-Azat et al. 2010; Doherty-Bone et al. 2013a). Research on the susceptibility of Cameroonian amphibians to infection by *Bd* could also take place, particularly the presence of skin peptides and microbiota that provide resistance to this fungal pathogen (Harris et al. 2006; Woodhams et al. 2007). This work will help determine the threat posed by *Batrachochytrium* pathogens to Cameroon’s amphibians.

Amphibian chytrid fungus is not the only pathogen that can cause declines in amphibians, with other diseases emerging (Duffus 2009). An enigmatic disease has been observed in the Lake Oku Clawed Frog (*Xenopus longipes*) since 2006, where many frogs have been found with lesions and necrotic limbs, but with the agent still unidentified (Blackburn et al. 2010a; Doherty-Bone et al. 2013b). Ranavirus has been found in one of these clinical specimens, but its presence needs to be verified and impacts on Cameroonian amphibians assessed. The role of disease for the conservation of Mt Oku’s amphibians remains uncertain and requires monitoring to detect emergences and declines so conservation action can be planned. Biosecurity should be practised by field workers to prevent the spread of novel and unknown pathogens to naïve amphibian populations (Phillott et al. 2010).

### Invasive alien species

Invasive alien species are organisms established by humans either intentionally or unintentionally outside of their natural range into a novel ecosystem that would not normally encounter that species. When they proliferate and spread without control, they can cause severe damage to native species, ecosystems and economies. Amphibians are particularly affected by invasive alien species, notably by introduced predators (especially fish) and pathogens (Kats and Ferrer 2003). On Mt Oku, the biggest threat from invasive alien species comes from aquaculture and the introduction of fish (possibly *Tilapia nilotica*) into Lake Oku (Gartshore 1986; Tinsley and Measey 2004). The latter has not yet happened but is still a looming threat to the endemic Lake Oku Clawed Frog, as stocking the lake is often discussed by the Oku community elders (TMD-B pers. ob.). Small-scale aquaculture already takes place in the form of numerous fish ponds around Mt Oku. The potential for fish to escape from ponds and invade Lake Oku have not been fully investigated, though all ponds are downhill from the lake, making passive escape following rainfall less imminent. However, other animals such as birds might spread organisms from these ponds to Lake Oku, especially fungal pathogens that can be carried by these animals (Garmyn et al. 2012). These ponds are often found to provide habitats for some anurans, especially *Xenopus eysoole*, but whether or not they breed in the presence of fish, or intensively stocked fish ponds is unknown. The domestic cat (*Felis domesticus*) is a particularly harmful invasive species in many regions (Lowe et al. 2000) and are beginning to become popular in Elak-Oku, owing to their value in controlling rodents in households. The potential of densities of cats to increase in the region and for them to establish populations is uncertain, but is potentially a new invasive alien species and should be monitored. Other invasive species that might threaten amphibians on Mt Oku are unknown. As with preventing new and unknown diseases, biosecurity should be practised by field workers (including tour guides) to prevent spread of new invasive alien species through inspecting, cleaning and drying equipment (Anderson et al 2015).

### Past, present and future conservation action

Conservation interventions relevant to amphibians on Oku have been indirect or patchy at best. The Birdlife International-Cameroon government Kilum-Ijim Forest Project has had some success in encouraging an environmental ethic in the local communities (Abott et al. 2001; TMD-B and VG pers. ob.) and reducing (but not stopping) the rate of forest loss and degradation (GIS Unit Royal Botanic Gardens Kew, downloaded 2016). The current status quo is the main forest block, the Kilum-Ijim Forest divided into 11 community forest management institutes, with the Kilum-Ijim Plantlife Sanctuary a protected government reserve that includes Lake Oku. This latter protected area is however infrequently patrolled by the forest guards, who seem to have a conflicting agenda with members of the community through prohibition of access to one part of the lake’s shore (TMD-B, pers. ob.). This hence occasionally affects local rights, tourism and perceptions of conservation by local people, who frequently ignore the access prohibition (TMD-B, pers. obs.). The Mbi Crater is also a government protected area, but again is infrequently patrolled, seems to have no management plan or mitigation against agrochemical run-off from the local tea estate. The forests around Big Babanki have no protection at all. The wetland habitat of Afua Swamp also has no protection, and the surrounding forest has been heavily degraded in the past decade (Khimal Peter, former Kilum-Ijim Project technician, pers. comm.), mostly by cattle grazing, which also threatens rare plants (Maisels et al. 2000).

Improving the future prospects for Mt Oku’s amphibians needs to build on these existing conservation interventions. This requires a concerted movement from sporadic surveys and preliminary appraisals of the human scale to taxonomic appraisals using modern methods, robust ecological and socio-economic study and active interventions (such as those listed in Smith and Sutherland 2014). Recent campaigns to collect better reference collections to enable molecular phylogenetic analysis to test taxonomic hypotheses have made significant advances (Zimkus 2009; Blackburn et al. 2010b; Doherty-Bone et al. 2011a; Zimkus and Gvoždík 2013). These have been coupled with engagement with the local communities raising awareness: public outreach meetings (Doherty-Bone 2011, 2015b), participation in traditional rituals to facilitate field surveys (Doherty-Bone et al. 2011b) and even naming of new species using indigenous languages (Zimkus 2009; Blackburn et al. 2010b; Zimkus and Gvoždík 2013; Evans et al. 2015). Work addressing threats directly also need to be focused into sustainable actions. For example, initial conservation action plans have been prepared for the habitats of three Critically Endangered amphibians: Lake Oku and the Oku Summit (Doherty-Bone, 2014, 2015). These action plans have been prepared in collaboration with local, regional, national and international representatives in Oku, as recommended in a newly accepted paradigm for environmental management in developing countries (Smith et al. 2009). These action plans have strong involvement with local stakeholders, particularly appropriate for the anurans restricted to the summit grasslands of Oku, where conflicts exist with livestock herders (Maisels et al. 2000). *Ex-situ* colonies of captive *Xenopus longipes* are being developed (Michaels et al. 2015; Tapley et al. 2016) following recommendations of the species’ 2004 IUCN assessment (Tinsley and Measey 2004), though it should be noted that *ex-situ* conservation projects can have limited effectiveness, particularly without adequate planning and collaboration *in situ* (Tapley et al 2015). Small steps have been made in addressing the threats to the Lake Oku Clawed Frog: firstly acquiring ecological data on this species; training local researchers; engaging and informing the community on issues such as the risks of fish introductions; and advocating the general importance of amphibians and environment on Oku. The impact of these conservation action plans have still to be assessed. Despite this, much work is needed to reinforce the long term prospects for this particular species and habitat.

The amphibian fauna of Mt Oku is particularly rich but threatened, and despite over 40 years of field research, more species may yet be described, more sections of the mountain need to be surveyed and the biology and ecology of these species to be adequately studied. The status of the many endemic and restricted-range species remains uncertain, though it is likely most are under pressure from multiple-threats for a changing region, country and globe. These threats can be addressed through protection of forest and high-elevation savanna with careful control of agrochemicals and biosecurity, with roles for international, national, regional and local conservation professionals and stakeholders alike.

## Appendix

**Table 1A.** Museum accession numbers for newly recorded taxa to Mount Oku, Cameroon. Abbreviations for museums: BM – Natural History Museum London, UK; NHMUK – Natural History Museum London UK, (new tag code); NMP6V – National Museum, Prague, Czech Republic

Species	Accession #	Year	Locality
*Arthroleptis adelphus*	NMP6V 73369	2005	Bamo Forest
	photo voucher	2006	Elemighong
Arthroleptis sp. aff. poecilonotus	NMP6V 73343/1-2,5	2005	Mejung
	NMP6V 74668	2010	
	BM20052327,2499-500	2012	Elemighong
Arthroleptis cf. perreti	NMP6V 73367, -35	2005	Mendong Buo
	BM20051932	2013	Emfveh-mi Forest
Astylosternus cf. diadematus	NMP6V 73373/1-2,	2005	Kedjom-Keku
	73374, 74674	2009	Mejung
*Asytlosternus montanus*	BM2008.442	2009	Afua Swamp
*Cardioglossa leucomystax*	NMP6V 73398/1-5	2005	Bamo Forest
*Leptodactylodon axillaris*	photo voucher	2006	Anyafouma Forest
*Leptodactylodon bicolor*	photo voucher	2006	Elemighong
	photo voucher	2015	Lui-Oku
*Leptopelis modestus*	ZMB 79624 (tadpole)	2012	Abu Forest
*Leptopelis notatus*	NMP6V 73394, 74608	2005	Mejung
		2009	
*Hyperolius ademetzi*	NMP6V 73384	2005	Lake Oku
*Hyperolius igbettensis*	BM20051994	2012	Mbi Crater
*Hyperolius nitidulus*	BM20051996-97	2012	Mbi Crater
Petropedetes sp. aff. parkeri	NMP6V 73389/1-2, 73391/1-2	2005	Kedjom-Keku, Mejung
*Phrynobatrachus calcaratus*	NMP6V 73399/1-12	2005	Bamo Forest
*Phrynobatrachus cricogaster*	NMP6V 73393/1-6	2005	Bamo Forest
*Phrynobatrachus natalensis*	photo voucher	–	Chuaku
	NHMUK201368,90-91	–	Lake Kuk
*Phrynobatrachus schioetzi*	NMP6V 73438	2005	Kedjom Keku (Mendong Buo)
	BM2008.537	2008	Klilum-Ijim Plantlife Sanctuary
	BM2008.551	2009	Afua Swamp
Phrynobatrachus sp. aff. werneri	BM2008.542	2009	Anyafouma Forest
Ptychadena cf. mascareniensis	BM2008.477	2009	Kissotin
	NMP6V 74606/1-2	2009	Mejung
Ptychadena cf. oxyrhynchus	NMP6V 74667/1-3	2010	Mejung
*Ptychadena taenioscelis*	BM2008.476	2009	Afua Swamp
*Sclerophrys latifrons*	NMP6V 73395/1-2	2005	Bamo Forest
*Sclerophrys maculata*	NMP6V 73378, 73380	2005	Bamo Forest, Mejung
*Sclerophrys regularis*	photo voucher	2006	Elemighong
*Sclerophrys superciliaris*	photo voucher	2005	Bamo Forest
*Sclerophrys villiersi*	NMP6V 74647	2009	Bambui
*Trichobatrachus robustus*	photo voucher	2006	Elemighong
	–	2008	Mbam-Oku
	–	2010	Kedjom-Keku
